# Predictive signatures of immune response to vaccination and implications of the immune setpoint remodeling

**DOI:** 10.1128/msphere.00502-24

**Published:** 2025-01-24

**Authors:** Irene Ramos

**Affiliations:** 1 Department of Neurology, Icahn School of Medicine at Mount Sinai, New York, New York, USA; 2 Department of Microbiology, Icahn School of Medicine at Mount Sinai, New York, New York, USA; 3 Precision Immunology Institute, Icahn School of Medicine at Mount Sinai, New York, New York, USA; University of Maryland School of Medicine, Baltimore, Maryland, USA

**Keywords:** vaccines, influenza, immunology

## Abstract

In 2020, I featured two articles in the “mSphere of Influence” commentary series that had profound implications for the field of immunology and helped shape my research perspective. These articles were “Global Analyses of Human Immune Variation Reveal Baseline Predictors of Postvaccination Responses” by Tsang et al. (Cell 157:499–513, 2014, https://doi.org/10.1016/j.cell.2014.03.031) and “A crowdsourced analysis to identify ab initio molecular signatures predictive of susceptibility to viral infection” by Fourati et al. (Nat Commun 9:4418, 2018, https://doi.org/10.1038/s41467-018-06735-8). From these topics, the identification of signatures predictive of immune responses to vaccination has greatly advanced and pivoted our understanding of how the immune state at the time of vaccination predicts (and potentially determines) vaccination outcomes. While most of this work has been done using influenza vaccination as a model, pan-vaccine signatures have been also identified. The key implications are their potential use to predict who will respond to vaccinations and to inform strategies for fine-tuning the immune setpoint to enhance immune responses. In addition, investigations in this area led us to understand that immune perturbations, such as acute infections and vaccinations, can remodel the baseline immune state and alter immune responses to future exposures, expanding this exciting field of research. These processes are likely epigenetically encoded, and some examples have already been identified and are discussed in this minireview. Therefore, further research is essential to gain a deeper understanding of how immune exposures modify the epigenome and transcriptome, influence the immune setpoint in response to vaccination, and define its exposure-specific characteristics.

## INTRODUCTION

The ability to predict how our bodies respond to potential perturbations or treatments has a tremendous impact on the future of medicine. In the field of vaccination, most of the population that fits some demographic criteria, such as age or specific medical conditions, are vaccinated with the same types of vaccines. However, there is great variability in people’s ability to generate protective responses after vaccination, which are determined by genetics as well as the history of environmental exposures. Thus, the possibility of predicting an individual’s response to vaccination lines up with the broadly pursued precision medicine (1), where vaccination strategies would be personalized given a person’s predicted ability to respond to immunization. Additionally, the identification of predictive signatures (or sets of genes) helps better understand the early molecular mechanisms that are associated with good vaccination responses.

The fundamental idea behind the studies investigating the prediction of outcomes is that human genotypic and phenotypic variation before vaccination explain a significant portion of the magnitude of the immune response. Since the important studies by Tsang et al. (2) and Fourati et al. (3) were featured in the mSphere of Influence commentary (4), there has been remarkable progress in the development of predictive models and identification of signatures associated with immune responses. One of the factors contributing to this rapid progress is the increased accessibility of single-cell transcriptomics assays, either alone or combined with other assay modalities such as surface protein staining. The increase in data complexity and modalities comes with bioinformatics challenges, which have also been actively tackled by multiple researchers in recent years. Here, I review the development of the field of prediction of influenza vaccination from pre-vaccination data. Then, I expand on how these findings relate to those from other vaccines and on the work that has been done to identify pan-vaccine predictors. These studies highlight the importance of the immune setpoint in the responsiveness to vaccination and suggest that this baseline can be modified by previous exposures such as virus infections or vaccinations. It is important to note that also several studies have developed early post-vaccination signatures, including influenza and other vaccines (5–10), which are not covered in this review.

## PREDICTIVE SIGNATURES OF GOOD RESPONSES TO INFLUENZA VACCINATION

The baseline populational variation encodes the key to predictions of immune responses to vaccinations. However, this variation can also obscure the predictive signals, and modeling techniques that reduce background noise are necessary. In the case of influenza vaccination, an important level of complexity to the baseline variation is added by the pre-existing immunity to previous influenza virus infections and vaccinations, which are known to greatly affect responses to vaccination (11, 12). Importantly, an early manuscript addressing this topic established predictive signatures measuring pre-existing antibodies to multiple hemagglutination (HA) peptides (13). Here, they identified two HA peptides which reactivity at baseline negatively correlated with the post-vaccination HA inhibition (HAI) response, calculated as the geometric mean titer for all strains in the vaccine. To overcome the problem of the interference of the pre-existing antibodies when using transcriptional or other immune data for the development of predictive models, researchers in this field developed a metric, named adjusted maximum fold change (adjMFC), to remove the previously observed nonlinear correlation between the maximum post-vaccination antibody titers and day 0 titers to each vaccine strain (2, 14). To evaluate the performance of predictive models, researchers use the area under the receiver operating characteristic curve (AUROC). This metric reflects how well a model predicts an outcome. A value of 1 indicates perfect accuracy, where the model predicts correctly 100% of the time. In contrast, a value of 0.5 indicates that the model’s predictions are no more accurate than random guessing, with only a 50% chance of being correct.

Furman et al. (13), in addition to the analysis of pre-existing antibody reactivity data, utilized a wide array of pre-vaccination data, including pre-existing T cell responses, serum proteins, cell-type frequencies, and microarray gene expression data, and developed multiple prediction models that could differentiate good responders (defined as subjects that seroconverted—fold-change HAI titers equal or higher than 4—for at least two vaccine strains) versus poor responders (seroconversion achieved for one strain or none) with high accuracy (up to AUROC = 0.84). The authors also studied the impact of age in the predictions and found that multiple predictors identified were age-associated. An important gene module that correlated with good responses was enriched in genes related to apoptosis including GSTP1, PIAS4, IL17D, and ZNF148, and was referred to as “APO” (Table 1). This module was also found to show decreased expression with age. In view of these results, the authors suggested that the identification of the APO module as predictive of influenza vaccination might be linked to the importance of apoptosis processes in the germinal center reactions, such as in selecting high‐affinity plasmablasts (15) or clearance of reactive B and T cells after the immune reaction takes place (16).

**TABLE 1:** Baseline transcriptomic signatures predictive of immune response to vaccination reported in previous studies

Name	Description	Gene signature	Reference(s)
APO module	Apoptosis related module enriched in high responders	LHFPL2, SLC27A3, C6orf192, TPRG1L, UBXN10, C20orf94, CSNK1G2, PAQR7, CHST13, DUSP3, PASK, BAMBI, RNF115, GSTP1, TRA2A, ANXA11, HOXD3, TMEM205, DYTN, FLJ38717, PIAS4, IL17D, GNL3L, PSMB10, WDR48, RPL3L, FAM23A, SNRPD3, LOC729590, VCP, ZNF148, FAM107B, KRBA1, EIF4G3, DCAF5, CFLAR, NAPRT1, VPRBP, FCHSD2	(13)
HIPC-Young-Sig	Predictive signature specific for young individuals	RAB24, GRB2, DPP3, ACTB, MVP, DPP7, ARPC4, PLEKHB2, ARRB1	(14)
TGSig	10-gene signature, associated with CD20^+^CD38^++^	C15orf57, LONP2, PAPSS2, EPHB1, ADAM12, SMC1A, RETN, ENPP1, CD101, C2orf63	(17)
IFN-I-DCact	Type I IFN and DC activation	ATF3, CCL8, CXCL10, DDX58, DDX60, DHX58, EIF2AK2, HERC5, IFI27, IFIH1, IFIT1, IFIT2, IFIT3, IRF7, LAMP3, MX2, OAS1, OAS3, OASL, PARP9, PLSCR1, PML, RSAD2, SERPING1, SP100, TAP1	(8, 17)
SLE-Sig	Leading edge in a module enriched for type I IFN-related genes	DDX60L, ANKFY1, IRF7,TRIM56, EPHB1, DHX58, STAT2, SP110, MOV10, NAGK, GBP1, TRIM22, IFIT2, OAS3, IRF9, DTNBP1, STAT1, XAF1, PARP9, IFIT1, BTN2A2, MX1, CXCL10, LGALS3BP, ARFIP1, UBE3B, EPSTI1, SERPING1, OAS1, DENND1A, TAP2, DDX60, DDAH2, FGR, PARP14, ODF3B, PARP12, CARD6, IFI27, IFIT3, IL1RN, FTSJD2, ACTA2, DISC1, GRAMD1B, GBP2, RSAD2, RNASE2, IFI44L, SCO2, IFIH1, CMPK2, ATG10, TYMP, SRGAP2, USP18, IFI35, IFI44, CLEC4A, SRBD1, TRIM25, MX2, LGALS9, CRIPT, LIMS1, OAS2, TUFT1, PANK2, TFE3, LMO2, BTN2A1, IFITM3, DMXL2, PSMB9, TOR1B, FYB, IFI6, MPZL2, SLFN12, GLA, ACOT9, ZNF333, RNPEP, ZNFX1, AIM2, ZCCHC6, HSH2D	(17)
CD40act	Related to stimulation of B cells by T cells through CD40-CD40L interactions	BUB1B, CCL22, CD58, CD59, DBI, DUSP4, FABP5, FDPS, FEN1, GAPDH, GARS, GRHPR, H2AFX, H2AFZ, HMGB2, HMMR, HPRT1, IMPDH2, LDHA, LDHB, LMNB1, LPXN, MCM2, MCM3, MCM6, MSH6, MTHFD2, MYBL2, NDC80, NME1, PAICS, PCNA, PKM, PRDX3, RFTN1, RGS10, SLAMF1, TK1, TOP2A, TPX2, TRAF1, TUBA1B, TXN, TYMS, UBE2S, VDAC1, WEE1, WSB2, ZWINT	(17–19)
Monocyte_ highresponder_ naturaladjuvant	Based on day 1 AS01 associated changes in monocytes	S100A11, S100A12, APOBEC3A, DDX60, DDX58, CREB5, TFEC, S100A9, GCA, FGL2, NACC2, KYNU, SLC16A3, MS4A4A, S100A8, FCGR3A, SAMHD1, TNFSF13B, C19orf59, CCR2, MARCO, P2RY13, RSAD2, SERPINA1, FPR1, FGR, PLSCR1, SIGLEC9, IFIH1, ACSL1, LMNB1, LRRK2, MNDA, PLBD1, KIAA0513, AQP9, SLC31A2, LILRB1, VCAN	(20)
mDC_highresponder_ naturaladjuvant	Based on day 1 AS01 associated changes in dendritic cells	SERPINA1, SLC31A2, TYMP, S100A11, MS4A4A, FCN1, LILRB2, PSMA7, CDC26, RB1, PSMB3, PLBD1, LMNB1, PPP2R5E, KYNU, S100A8, S100A9, PSMB6, RBX1	(20)
Hep-B-over65	HepB vaccination response predictor in older adults	HP/HPR, CR593799, SYDE2, DQ145726, KIF18B, EFHC2, TSPAN13, CD20, AMMECR1, BX537613, BANK1, SPTBN1, SPTBN1, CD20, IGHG1	(21)
PanVac_Sig	14 genes contributing to the majority (>50%) of pan-vaccine classifier predictions	KCNJ2, UTY, CNTNAP2, PTGS2, MAPK8IP1, LTC4S, ZNF124, EREG, CASP5, EGR1, CXCL10, ZNF248, DDX3Y, CCL20	(22)
IHM transcriptional surrogate	Immune health metric blood transcriptional surrogate	SLC16A10 NDRG2, AK5, CD7, RNFT2, PHC1, MAN1C1, FAM102A, RCAN3, EIF3H, RPS17, RPS5, PLXDC1, TESPA1, SGK223, EIF3L, ANAPC16, CD27, NPAT, ID3, RACK1, APEX1, GCNT4, FCMR, TCF7, KLHL3, AXIN2, LY9, RPS25, LDHB, PKIA, RPL3, N6AMT1, GAL3ST4, SSBP2, CD1C, LEF1, RPL7, PIK3IP1, GPRASP1, ABI2, APBB1, SPTBN1, GPA33, CCR9, BCKDHB, SCAI, RPL4, NOG, TCEA3, ETS1, LDLRAP1, GPR183, ZNF548, ZNF91, NPM1, MSANTD2, KAT6B, SLC7A6, DCHS1, OXNAD1, RPS2, RPL7A, GRAP, RPL23A, RPL10A, RPS3, FAM175A, RPL29, EEF2, EDAR, ABLIM1, MBLAC2, CCR7, ZNF573, CAMK4, LRRN3, MAGI3, RPLP2, ZIK1, NT5E, FUT8, ZNF101, RPL34, RPS20, FOXP1, ZNF550, TSPYL2, ATP6V0E2-AS1, GRPEL2, MGC57346, SEPT1, PRKACB, AGMAT, RPL11, MYC, ZZZ3, RPL5, CTRL, NTNG2, AP5B1, PDCD1LG2, DOCK4, S100A9, BACH1, FAM8A1, SECTM1, S100A8, TYMP, HK3, IL1RN, MYD88, REC8, ALPK1, SAT1, PRKCD, SLC26A8, PARP9, LMNB1, RELT, TAP1, JAK2, BRI3, GBP2, PLEKHO2, ETV7, ODF3B, SIGLEC5, CEACAM1, CARD16, ZBP1, DDX60L, APOL2, CD63, TNFAIP6, KCNJ2, ANKRD22, SCARF1, SEMA4A, DNAJC5, SQRDL, HELZ2, GADD45B, FAS, HSPA6, PIK3AP1, CLEC7A, SERPING1, OR52K2, ITPRIP	(23)

Using microarray data and flow cytometry cell-type frequencies from peripheral blood mononuclear cells (PBMCs) of 63 influenza vaccinees, Tsang et al. (2) found that cell frequency information by itself had predictive value, while the pre-vaccination transcriptomic data alone did not perform well at predicting post-vaccination titers in these data sets. Among cell types predictive of high adjMFC (calculated using neutralization assay titers; high responders and low responders were defined as those with adjMFC higher than the 80th percentile value and lower than the 20th percentile value, respectively), they found subpopulations of CD20^+^ B cells with high expression of CD38. A subsequent multicohort study by the Human Immunology Project Consortium (HIPC)-Center for Human Immunology (CHI) Signatures Project Team (14) was able to identify a transcriptomic signature that was associated with increased magnitude of antibody responses (adjMFC, calculated with HAI titers for all cohorts but one, which reported neutralization assay titers) in young individuals, and included nine genes that were validated in independent cohort studies (Table 1, HIPC-Young-Sig). The signature identified high responders (those with adjMFC higher than the 70th percentile value) versus low responders (those with adjMFC lower than the 30th percentile) with an AUROC = 0.83–1 in discovery cohorts and an AUROC = 0.79 in validation cohorts. Only one of these genes (RAB24) could be identified in single cohort analyses. This highlights the added power of incorporating multiple cohorts, which substantially increases the sample size. This is particularly important in this context, given that the baseline expression patterns that are linked to post-vaccination immune responses are expected to be subtle, and that there is high inter-individual variability in the antibody response to vaccination. The predictive power of this signature was partially due to differences in cell proportions, as its performance decreased after adjusting for cell-type proportions, while still showing modest predictive performance (AUROC = 0.68). BCR signaling and platelet activation blood transcription modules (BTMs) (8) were significantly different between high and low responders, even after considering cell-type proportion differences. An important observation in this study was that the identified signature was only associated with vaccine-induced antibody responses in young individuals, but not in old individuals. Interestingly, there was an inverse correlation in the effects of the identified signatures in antibody responses between young and older cohorts. This underlines the importance of considering age in vaccination prediction models, given the potential differences in the drivers of improved antibody production at different ages.

Transcriptomic signatures are far more convenient to use across studies than flow cytometry data due to the high technical variability among labs in the flow cytometry assays, which is influenced by factors such as antibody panel design and gating strategies. Following up on the cell-type frequencies as predictors of pre-vaccination signatures by Tsang et al. (2), the same group developed a 10-gene transcriptional signature (TGSig) that correlated with the frequencies of CD20^+^CD38^++^ B cell subtypes, and validated this signature as a predictor of not only influenza virus vaccination in several cohorts (AUROC = 0.80–0.88), but also of yellow fever vaccination (AUROC = 0.86) utilizing independent data sets (17). They also examined if TGSig was associated with systemic lupus erythematosus (SLE), an autoimmune disorder where at moments of disease activity there might be processes triggered that are similar to those occurring during vaccination. Interestingly, they found that, in a group of patients where disease activity was associated with a plasma cell/plasmablast signature, TGSig evaluated at clinically quiescent times was correlated with the disease-activity-associated change in plasmablast score (DaCP). Next, they assessed if other SLE-associated signatures could be predictive of vaccination responses and found a type I interferon (IFN) signature module during clinical quiescence that could be prognostic of disease activation in patients with plasmablast-associated flares. These type I IFN-related modules reflect overlap between SLE and pre-vaccination transcriptional profiles that promote antibody responses, and further analysis showed aspects related to type I IFN response and DC activation, which were narrowed down to a new signature including 26 genes named IFN-I-DCact (Table 1). To further map the predictive signatures to cell types, the authors incorporated the use of Cellular Indexing of Transcriptomes and Epitopes sequencing (CITE-seq) and applied it to PBMCs from healthy influenza vaccinees, including high and low responders. CITE-seq enables the precise identification of immune cell types, traditionally characterized by flow cytometry, while also providing an in-depth measurement of their transcriptomic cell states. Thus, they found that TGSig was significantly higher in plasmacytoid dendritic cells (pDCs) from high responders as compared to low responders, which are known to activate T cells that stimulate B cells via CD40L^−^CD40 interaction. To assess the involvement of this process in a favorable pre-vaccination setpoint, an additional signature was generated derived from previous literature and was named CD40act (Table 1). The CD40act signature was found to be increased in high responders in memory CD4^+^ T cells at baseline and correlated with the frequency of the originally identified CD20^+^CD38^++^ B cells. Therefore, this study, which combined multiple previously reported transcriptomic data sets and an original CITE-seq data set, allowed to establish a working model where, in high responders, there are elevated levels of pDC activation, potentially leading to type I IFN release and high levels of activation of T cell and B cell subpopulations. This, in turn, would result in more pronounced plasmablast/plasma cell differentiation upon antigen encounter during vaccination.

The recent study by Mule et al. (20) provided further insights into the transcriptomic signatures that are associated with improved responses to vaccination using CITE-seq combined with a multilevel modeling framework to integrate human population, temporal, and cell-type-specific transcriptional variability. Here, the authors first defined early immune responses that are induced by an AS03-adjuvanted, inactivated, H5N1 vaccine early after vaccination, which are expected to be associated with more efficient responses. In monocytes and myeloid dendritic cells (mDCs), AS03-adjuvanted vaccine induced increased expression levels of innate immune receptors, as well as of chemotaxis and IFN-related genes, suggesting that AS03 increases the ability of antigen sensing and presentation of myeloid cells. To associate baseline signatures with the level of responsiveness to an unadjuvanted vaccine and to understand how different cell subsets correlate with each other, Mule et al. identified 81 baseline setpoints that formed a correlated network of cell-type transcriptional phenotypes. This suggests that the immune setpoint associated with antibody responses is attributed to coordinated transcriptional states across multiple cell types, rather than to individual cell types. Some of the nodes identified in this analysis were related to the day 1 AS03 associated signatures, including ISGs in CD14 monocytes, Fc receptor genes, phagocytosis genes, and second messenger signaling molecules, suggesting a resemblance between high responders’ baseline setpoint and early adjuvant-mediated transcriptional changes. Next, they refined two novel transcriptomic signatures, referred to as “naturally adjuvanted” in monocytes and mDCs (Table 1). These signatures were enriched in high responders compared to low responders at baseline in subjects that received an unadjuvanted seasonal influenza vaccine, indicating that high responders possess a naturally adjuvanted immune setpoint that mirrors the early immune responses observed following adjuvanted vaccination.

Another study published by Sevim Bayrak et al. (24) analyzed pre-vaccination samples from recipients of the seasonal influenza vaccine and integrated transcriptomics, proteomics, metabolomics, and glycomics data. Five clusters were identified and were associated with different levels of gene expression related to innate immune pathways or adaptive immune pathways. Surprisingly, the cluster with the highest representation of adaptive immunity features showed the lowest levels of circulating IgA, but increased post-vaccination titers of the B/Yamagata vaccine strain. The highest levels of pre- and post-vaccination H3N2 IgA antibodies were found in the group with the highest representation of innate immune pathways (i.e., response to virus and type I IFN signaling pathways). Overall, in this study, while there were associations between the five phenotypic clusters identified with pre-existing levels of total IgA and HAI levels for some of the vaccine strains, there was high variation in the post-vaccination responses and predictive patterns could not be identified.

Therefore, multiple strategies have been employed to identify a series of predictive signatures of influenza vaccination, which are summarized in Table 1. These approaches involved the integration of data from various cohorts, as well as the application of advanced multimodal single-cell technologies such as CITE-seq. By leveraging these innovative methods, researchers have been able to uncover gene signatures and immune processes that, when assessed at baseline, correlate with or predict post-vaccination antibody titers.

## PREDICTION OF RESPONSES TO OTHER VACCINES AND IDENTIFICATION OF PAN-VACCINE PREDICTORS

As described above, the work on influenza vaccines has yielded multiple transcriptomic signatures that predict immune responses to influenza vaccination. Other vaccines have also been investigated in this context, showing both coherent and conflicting results compared to the influenza vaccine studies. Therefore, to understand the challenges to predict universal immune responses to vaccination, it is important to first review the findings for different vaccines.

A study analyzing predictors of immune response to vaccination, which aimed to understand how aging is associated with vaccine hypo-responsiveness, investigated a cohort of older adults vaccinated against hepatitis B (recombinant antigen) (21). In this study, the antibody response was quantified as the serum concentration of Hepatitis B surface antigen (HBsAg)-specific total antibodies, and subjects were classified as responders (values ≥5 mIU/mL), or poor responders (values <5 mIU/mL). Interestingly, increased memory B cell frequencies (IgG^+^ memory B cells) were predictive of enhanced vaccination responses, similar to findings by Tsang et al. in the case of influenza vaccinees (2). They also found that expression of genes related to B cell function at baseline was associated with higher responses post-vaccination. However, a module of pro-inflammatory genes, which included important innate immune genes such as IRF5 or MYD88, was associated negatively with adaptive immune responses, which contrasts with previous findings in multiple influenza vaccine studies. It is important to note that the multicohort influenza vaccine study in 2017 (14) found inverse patterns in young and older adults, and the signature they developed in younger adults did not predict immune responses in older adults. Disparities in sample sizes in the older cohorts of both studies are unlikely to explain these differences, since they were in the same range (144 and 157 subjects in the hepatitis B and in the influenza vaccine studies, respectively). However, it is important to note that, while the hepatitis B vaccine study was performed in an antigenic naïve population, in the influenza vaccine studies most of the population have been most likely previously exposed to multiple influenza virus infections or vaccines. A signature of 15 genes (Table 1) was derived from this study and was shown to predict the magnitude of the antibody response to vaccination with an AUROC of 0.62 (21). Interestingly, a study that analyzed the baseline transcriptomic profile in malaria vaccinees (RTS,S/AS01), found that monocytes and DCs BTMs were inversely correlated with protection (25), which contrasts with findings from influenza vaccine studies as well. In this case, protection was evaluated as risk of acquiring malaria infection. It is possible that the different results are due to different mechanisms of protection as compared to influenza vaccines, which in addition to antibody responses, also involve antigen-specific Th1 cytokines (26).

Therefore, identifying a universal signature predictive of vaccination responses is a complex task given the heterogeneity across vaccine platforms and antigens, which may drive immune activation through diverse molecular and immunological mechanisms, as well as correlates of protection for different diseases. In order to tackle this problem, an important study utilized public transcriptomics data from whole blood or PBMCs across 13 different vaccines (22) that were available through Immport and compiled as the “Immune Signatures Data Resource” accessible through ImmuneSpace (27). The vaccines included a broad range of platforms and pathogens: Ebola (recombinant viral vector), hepatitis B (inactivated), HIV (recombinant viral vector), influenza (inactivated and live virus), malaria (recombinant protein), meningococcus (conjugate and polysaccharide), pneumococcus (polysaccharide), smallpox (live virus), tuberculosis (recombinant viral vector), varicella zoster (live virus), and yellow fever (live virus). By performing unsupervised clustering of the pre-vaccination samples, they identified three endotypes with different gene expression patterns. One of them, named “inflam.hi,” showed enhanced expression of monocytes and DC markers, ISGs, and pro-inflammatory genes compared to the other two, named “inflam.mid” and “inflam.lo.” Among these, there were genes important for pattern recognition (such as toll-like receptors or RIG-I), IFN receptors, IFN-related transcription factors, pro-inflammatory cytokines and proteins involved in IFN and cytokine downstream signaling. Participants with the “inflam.hi” endotype showed higher antibody responses across all vaccines included in the study. However, this difference was only significant for the influenza vaccines when comparisons were made for each vaccine individually. Next, they built a classifier using this pan-vaccine data, which achieved an AUROC of 0.62 as estimated by tenfold cross-validation, showing some predictive value when all vaccines were considered. Fourteen genes contributed to the majority of the prediction capability of the classifier (PanVac-Sig, Table 1), and did not overlap with previously published signatures. This 14-gene signature showed significantly better identification than random in multiple vaccine data sets, including influenza, yellow fever, and pneumococcus vaccines. However, six previous signatures that were evaluated showed better performance for the vaccine types they were trained on but less on the others. To assess the cell-type contribution to the inflammatory signature associated with pan-vaccination responsiveness, the authors selected 50 inflammatory genes that overlapped between the “inflam.hi” group identified in the unsupervised clustering analysis and the supervised classifier, and evaluated their cell-type distribution using the CITE-seq data reported by Kotliarov et al. (17). Interestingly, and in agreement with findings by Mule et al. (20), monocytes and mDCs showed enrichment of inflammatory genes associated with good responses to influenza vaccination. In a later study, Sparks et al. (23) developed a human immune health metric (IHM) utilizing transcriptomics, serum protein data, blood cell-type frequency, and clinical data from patients with multiple monogenic conditions affecting immunological pathways, and healthy donors. The IHM signature was proven to be a strong marker of immune health and showed an inverse correlation with age. To improve generalizability of the signature, authors derived IHM blood transcriptional and circulating protein surrogate signatures. The IHM transcriptional signature (Table 1) was evaluated as a potential baseline predictor of immune responses to vaccination on the same data utilized by previous pan-vaccination studies (10, 22). The IHM transcriptional surrogate was predictive of vaccine antibody responses in subjects older than 50 years old, but did not perform well in younger individuals, as opposed to the previously described HIPC-Young-Sig (Table 1) (14). Therefore, IHM is a metric of immune health that predicts age-dependent responsiveness to vaccination.

In conclusion, the results from the ambitious studies pursuing the identification of pan-vaccination predictive signatures have shown that there may be common pre-vaccination features associated with vaccine efficacy. Of note, overall improved performance was obtained for influenza vaccines, which is the vaccine type with higher current data availability. Future research, potentially involving a higher number of participants across other vaccine types and more single-cell data sets, will allow for the further definition and determination of these signatures, leading to models with improved performance that could help identify individuals with globally low responses to vaccination.

## RESHAPING OF THE IMMUNE SETPOINT THROUGH EXPOSURES

The mechanisms by which immune setpoints are established remain unclear. It is well described that age (28, 29), sex (30, 31), and race (32) influence immune responses to vaccination. Genetics also play an important role (33), but a study of healthy twins found that non-heritable traits explain a large fraction of the variation in immune measurements, and did not find a heritable component in antibody responses to seasonal influenza vaccination (34). Therefore, it is probable that previous exposures reshape our immune system, contributing to the variation in immune responsiveness to vaccination. Importantly, it is becoming increasingly clear that acute respiratory infections and some vaccinations induce epigenetic remodeling of immune cells (35), which might result in altered transcriptional baselines as well as changes in the level of induction of innate and adaptive immune responses upon a subsequent perturbation.

Influenza virus vaccination induces epigenetic signatures that can persist for several weeks or even months in humans. The study by Wimmers et al. (36) found that monocytes and DCs from influenza vaccinees had reduced levels of H3K27ac, which indicates an overall reduction of transcription levels. Reduced chromatin accessibility of loci targeted by AP-1 transcription factor and lower responsiveness to toll-like receptor stimulation *in vitro* with respect to the pre-vaccination time point were also identified. Interestingly, an H5N1 inactivated vaccine administered with AS03 adjuvant, in addition to reduced chromatin accessibility of AP-1 target genes, also induced increased accessibility in IFN response factor (IRF) foci in myeloid cells, which resulted in enhanced antiviral responses (36). Along the same lines, a recent study found that two doses of the inactivated H5N1 influenza vaccine, either administered with or without AS03 adjuvant, induced transcriptional and epigenetic changes in classical CD14^+^ monocytes and CD8^+^ naive-like T cells that lasted for at least 100 days (37). Importantly, these persistent post-vaccination signatures were also found to be elevated at baseline in subjects with high antibody responses to vaccination. An additional study that analyzed blood transcriptomic profiles before and after the trivalent inactivated vaccine adjuvanted with MF59 found multiple blood modules altered by influenza vaccination at day 28 after vaccination in children 15–24 months old (38). Enhanced innate immune pathways included genes enriched in monocytes and activated DCs, toll-like receptor and inflammatory signals, and antigen presentation.

We have recently found that influenza virus infection in humans also induces remodeling of the epigenomic landscape that can be identified four weeks after infection in blood cells (39). Interestingly, some of our observations, including alterations in components of AP-1 gene regulation and IFN pathways in innate immune cells, resemble those found by Wimmers et al. (36) in the context of influenza vaccination. Specifically, the post-influenza virus infection signature is characterized by decreased expression of the genes encoding for the AP-1 transcription factor and its target genes in innate immune cells, and decreased accessibility of AP-1 targets and interleukin-related genes (39). In addition, we found that influenza virus infection also results in altered expression of genes involved in the MAP kinase pathways, and increased accessibility of IFN-related gene promoters (39). In a different study, we found that SARS-CoV-2 infection induces a long-term remodeling of the blood DNA methylation profile, which included a high representation of type I IFN-related genes, and resembled the one found in SLE patients (40). Forty-four of the 86 genes of SLE-Sig (17) were found among the ones with differentially methylated regions in our COVID-19 study (40). Importantly, a recent study found that prior mild SARS-CoV-2 infection (on average 6 months since acute disease) resulted in increased antibody responses to influenza vaccines in recovered males, but not in recovered females (41). In this study, the process was thought to be mediated by (i) elevated pre-vaccination signatures of CD8^+^GPR56^+^ “virtual memory”-like T cells, which produce enhanced IFNgamma after stimulation by IL-15; and (ii) increased production of IL-15 in monocytes, which activates virtual memory T cells.

The use of antibiotics has been also shown to induce transcriptomic changes in blood and affect the immune response to influenza vaccination through the alteration of the gut microbiome in subjects with low levels of pre-existing immunity (42). Specifically, reduced neutralizing activity against the H1N1 vaccine strain of the seasonal influenza vaccine, and reduced HA (H1N1)-specific IgG1 antibodies were found in subjects with antibiotic treatment as compared to controls. The authors also evaluated the impact of antibiotics in the blood transcriptome and cell frequency, and identified increased inflammatory signaling, activation of DCs, and increased frequency of mDCs and pDCs. Interestingly, and opposed to what was found in the context of influenza infection and vaccination, they identified activation of pathways associated with AP-1 (42).

**Fig 1:**
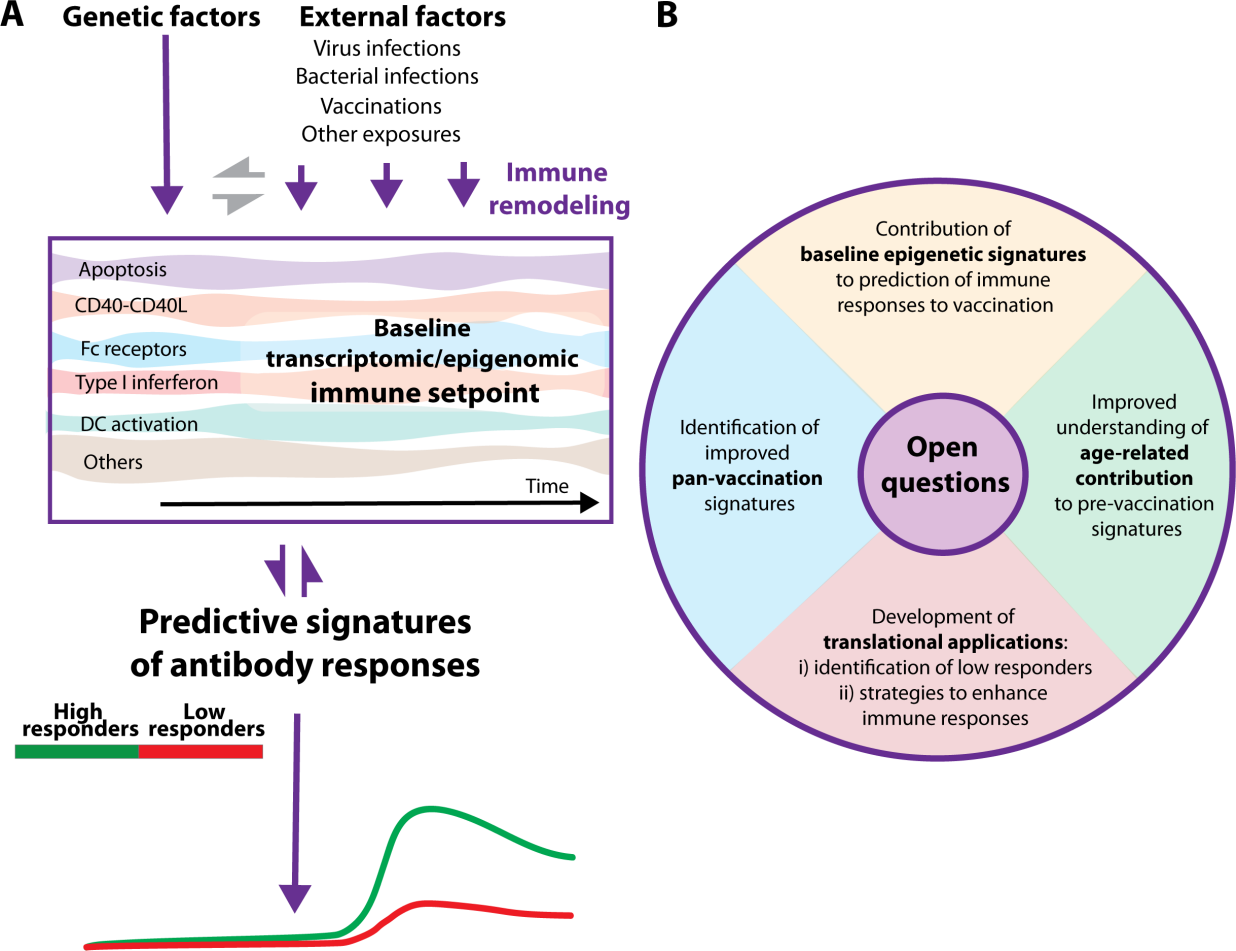
Predictive signatures of immune response to vaccination. (**A**) Implications of the immune setpoint remodeling through exposures in the magnitude of the antibody response to vaccination and in the development of predictive signatures. (**B**) Graphic representation of open questions in this field.

Overall, the findings in this field suggest that exposures such as acute respiratory infections or vaccinations remodel the innate immune system, as determined by the analysis of human blood cells using various assay modalities. Continuous exposure to various external factors, along with interactions with genetic factors, modifies the immune setpoint, including previously identified and yet unknown signatures that may influence responsiveness to future vaccinations (Fig. 1).

## OPEN QUESTIONS AND PROSPECTIVE RESEARCH DIRECTIONS

As evidenced here, the field of prediction of immune responses to vaccination has tremendously evolved in the last few years. Multiple determinants of successful influenza immunization in humans have been identified, including gene signatures and cell types, and pan-vaccination signatures with modest predictive performance have been developed. The increased availability of human vaccination data sets, and of single-cell platforms utilizing multiple-omics modalities (protein labeling, RNA-sequencing, and ATAC-seq) will continue to enable access to complex molecular and immunological data, contributing to the further development of this field.

A limited amount of work has been done utilizing epigenetic information in this context. However, progress is expected to be supported by the incorporation of epigenetics data, which potentially encode additional baseline information valuable for determining a subject’s ability to develop effective antibody responses to vaccination. Recent studies using DNA methylation data found multiple associations between DNA methylation profile and immune responses to influenza virus vaccination (43, 44). The sites with predictive DNA methylation signatures were enriched for genes involved in antiviral responses, such as the RIGI-I signaling pathway, and binding sites for the transcription factor BRD4 (43). In addition, these studies also found that influenza vaccination induces changes in the DNA methylation profile, supporting the idea that exposures such as vaccination contribute to epigenetic remodeling of the immune setpoint.

A translational application of identifying signatures predictive of immune responses to vaccination, in addition to tailoring strategies for low responders, is the implementation of therapies to enhance an individual’s ability to respond to vaccination. However, an important consideration when establishing strategies to modulate the immune setpoint for improved vaccine responses is reactogenicity. The work by Kazmin et al. found a type I IFN signature that, in addition to being associated with improved antibody responses, was also correlated with local immunogenicity (38). In addition, as found by Kotliarov et al. (17), there are similarities between some predictive signatures of antibody responses and autoimmune disease activity. This underscores the need to fine-tune specific components of the immune system to achieve effective antibody responses to vaccination, using carefully controlled strategies that minimize vaccine reactogenicity.

An additional aspect of this field that needs further investigation is the association of the profiles predictive of immune responses with age. The baseline transcriptional profiles associated with vaccine responses by HIPC-CHI in 2017 (14) in young individuals are not predictive in older adults and, in this particular study, they are inversely correlated. In addition, the study by Furman et al. (13) identified an age-associated apoptosis signature predictive of vaccination, and Spark et al. (23) identified a signature that is predictive in older adults but not in younger adults. Nevertheless, immune mechanisms in aging-related decreased vaccination responses are not well understood and require future investigations.

Therefore, the immune system is a continuously evolving network shaped by genetics and external exposures. These processes determine the immunological state of the immune system at homeostatic levels, and ongoing exposures fine-tune the system toward optimal or suboptimal immune responses to vaccination (Fig. 1). While most studies have used transcriptomic data due to its greater availability, epigenetic data have the potential to provide highly meaningful insights for improving immune responses and are increasingly being incorporated into multiple studies. Given our current understanding of this field and the valuable contributions from all the authors cited in this review, strategies to modify and enhance basal immune states in preclinical models are expected to be included in future research portfolios.

## References

[1] U.S. Food and Drug Administration CfDaRH. 2018. Precision medicine. Retrieved 27 Aug 2024.

[2] Tsang JS, Schwartzberg PL, Kotliarov Y, Biancotto A, Xie Z, Germain RN, Wang E, Olnes MJ, Narayanan M, Golding H, Moir S, Dickler HB, Perl S, Cheung F, Baylor HIPC Center, CHI Consortium. 2014. Global analyses of human immune variation reveal baseline predictors of postvaccination responses. Cell 157:499–513. doi:10.1016/j.cell.2014.03.031 24725414 PMC4139290

[3] Fourati S, Talla A, Mahmoudian M, Burkhart JG, Klén R, Henao R, Yu T, Aydın Z, Yeung KY, Ahsen ME, et al.. 2018. A crowdsourced analysis to identify ab initio molecular signatures predictive of susceptibility to viral infection. Nat Commun 9:4418. doi:10.1038/s41467-018-06735-8 30356117 PMC6200745

[4] Ramos I. 2020. mSphere of influence: predicting immune responses and susceptibility to influenza virus-may the data be with you. mSphere 5:e00085-20. doi:10.1128/mSphere.00085-20 32188748 PMC7082138

[5] Bartholomeus E, De Neuter N, Meysman P, Suls A, Keersmaekers N, Elias G, Jansens H, Hens N, Smits E, Van Tendeloo V, Beutels P, Van Damme P, Ogunjimi B, Laukens K, Mortier G. 2018. Transcriptome profiling in blood before and after hepatitis B vaccination shows significant differences in gene expression between responders and non-responders. Vaccine (Auckl) 36:6282–6289. doi:10.1016/j.vaccine.2018.09.001 30205979

[6] Querec TD, Akondy RS, Lee EK, Cao W, Nakaya HI, Teuwen D, Pirani A, Gernert K, Deng J, Marzolf B, Kennedy K, Wu H, Bennouna S, Oluoch H, Miller J, Vencio RZ, Mulligan M, Aderem A, Ahmed R, Pulendran B. 2009. Systems biology approach predicts immunogenicity of the yellow fever vaccine in humans. Nat Immunol 10:116–125. doi:10.1038/ni.1688 19029902 PMC4049462

[7] Tan Y, Tamayo P, Nakaya H, Pulendran B, Mesirov JP, Haining WN. 2014. Gene signatures related to B-cell proliferation predict influenza vaccine-induced antibody response. Eur J Immunol 44:285–295. doi:10.1002/eji.201343657 24136404 PMC3973429

[8] Li S, Rouphael N, Duraisingham S, Romero-Steiner S, Presnell S, Davis C, Schmidt DS, Johnson SE, Milton A, Rajam G, Kasturi S, Carlone GM, Quinn C, Chaussabel D, Palucka AK, Mulligan MJ, Ahmed R, Stephens DS, Nakaya HI, Pulendran B. 2014. Molecular signatures of antibody responses derived from a systems biology study of five human vaccines. Nat Immunol 15:195–204. doi:10.1038/ni.2789 24336226 PMC3946932

[9] Nakaya HI, Wrammert J, Lee EK, Racioppi L, Marie-Kunze S, Haining WN, Means AR, Kasturi SP, Khan N, Li GM, McCausland M, Kanchan V, Kokko KE, Li S, Elbein R, Mehta AK, Aderem A, Subbarao K, Ahmed R, Pulendran B. 2011. Systems biology of vaccination for seasonal influenza in humans. Nat Immunol 12:786–795. doi:10.1038/ni.2067 21743478 PMC3140559

[10] Hagan T, Gerritsen B, Tomalin LE, Fourati S, Mulè MP, Chawla DG, Rychkov D, Henrich E, Miller HER, Diray-Arce J, Dunn P, Lee A, Human Immunology Project Consortium (HIPC), Levy O, Gottardo R, Sarwal MM, Tsang JS, Suárez-Fariñas M, Sékaly R-P, Kleinstein SH, Pulendran B. 2022. Transcriptional atlas of the human immune response to 13 vaccines reveals a common predictor of vaccine-induced antibody responses. Nat Immunol 23:1788–1798. doi:10.1038/s41590-022-01328-6 36316475 PMC9869360

[11] Andrews SF, Kaur K, Pauli NT, Huang M, Huang Y, Wilson PC. 2015. High preexisting serological antibody levels correlate with diversification of the influenza vaccine response. J Virol 89:3308–3317. doi:10.1128/JVI.02871-14 25589639 PMC4337521

[12] Knight M, Changrob S, Li L, Wilson PC. 2020. Imprinting, immunodominance, and other impediments to generating broad influenza immunity. Immunol Rev 296:191–204. doi:10.1111/imr.12900 32666572

[13] Furman D, Jojic V, Kidd B, Shen-Orr S, Price J, Jarrell J, Tse T, Huang H, Lund P, Maecker HT, Utz PJ, Dekker CL, Koller D, Davis MM. 2013. Apoptosis and other immune biomarkers predict influenza vaccine responsiveness. Mol Syst Biol 9:659. doi:10.1038/msb.2013.15 23591775 PMC3658270

[14] Avey S, Cheung F, Fermin D, Frelinger J, Gaujoux R, Gottardo R, Khatri P, Kleinstein SH, Kotliarov Y, Meng H, et al.. 2017. Multicohort analysis reveals baseline transcriptional predictors of influenza vaccination responses. Sci Immunol 2. doi:10.1126/sciimmunol.aal4656 PMC5800877 28842433

[15] Smith KG, Light A, O’Reilly LA, Ang SM, Strasser A, Tarlinton D. 2000. Bcl-2 transgene expression inhibits apoptosis in the germinal center and reveals differences in the selection of memory B cells and bone marrow antibody-forming cells. J Exp Med 191:475–484. doi:10.1084/jem.191.3.475 10662793 PMC2195819

[16] Nagata S. 1999. Fas ligand-induced apoptosis. Annu Rev Genet 33:29–55. doi:10.1146/annurev.genet.33.1.29 10690403

[17] Kotliarov Y, Sparks R, Martins AJ, Mulè MP, Lu Y, Goswami M, Kardava L, Banchereau R, Pascual V, Biancotto A, Chen J, Schwartzberg PL, Bansal N, Liu CC, Cheung F, Moir S, Tsang JS. 2020. Broad immune activation underlies shared set point signatures for vaccine responsiveness in healthy individuals and disease activity in patients with lupus. Nat Med 26:618–629. doi:10.1038/s41591-020-0769-8 32094927 PMC8392163

[18] Gricks CS, Zahrieh D, Zauls AJ, Gorgun G, Drandi D, Mauerer K, Neuberg D, Gribben JG. 2004. Differential regulation of gene expression following CD40 activation of leukemic compared to healthy B cells. Blood 104:4002–4009. doi:10.1182/blood-2004-02-0494 15161673

[19] Shimabukuro-Vornhagen A, Zoghi S, Liebig TM, Wennhold K, Chemitz J, Draube A, Kochanek M, Blaschke F, Pallasch C, Holtick U, Scheid C, Theurich S, Hallek M, von Bergwelt-Baildon MS. 2014. Inhibition of protein geranylgeranylation specifically interferes with CD40-dependent B cell activation, resulting in a reduced capacity to induce T cell immunity. J Immunol 193:5294–5305. doi:10.4049/jimmunol.1203436 25311809

[20] Mulè MP, Martins AJ, Cheung F, Farmer R, Sellers BA, Quiel JA, Jain A, Kotliarov Y, Bansal N, Chen J, Schwartzberg PL, Tsang JS. 2024. Integrating population and single-cell variations in vaccine responses identifies a naturally adjuvanted human immune setpoint. Immunity 57:1160–1176. doi:10.1016/j.immuni.2024.04.009 38697118

[21] Fourati S, Cristescu R, Loboda A, Talla A, Filali A, Railkar R, Schaeffer AK, Favre D, Gagnon D, Peretz Y, Wang I-M, Beals CR, Casimiro DR, Carayannopoulos LN, Sékaly R-P. 2016. Pre-vaccination inflammation and B-cell signalling predict age-related hyporesponse to hepatitis B vaccination. Nat Commun 7:10369. doi:10.1038/ncomms10369 26742691 PMC4729923

[22] Fourati S, Tomalin LE, Mulè MP, Chawla DG, Gerritsen B, Rychkov D, Henrich E, Miller HER, Hagan T, Diray-Arce J, Dunn P, Human Immunology Project Consortium (HIPC), Levy O, Gottardo R, Sarwal MM, Tsang JS, Suárez-Fariñas M, Pulendran B, Kleinstein SH, Sékaly R-P. 2022. Pan-vaccine analysis reveals innate immune endotypes predictive of antibody responses to vaccination. Nat Immunol 23:1777–1787. doi:10.1038/s41590-022-01329-5 36316476 PMC9747610

[23] Sparks R, Rachmaninoff N, Lau WW, Hirsch DC, Bansal N, Martins AJ, Chen J, Liu CC, Cheung F, Failla LE, et al.. 2024. A unified metric of human immune health. Nat Med 30:2461–2472. doi:10.1038/s41591-024-03092-6 38961223 PMC12183718

[24] Sevim Bayrak C, Forst CV, Jones DR, Gresham DJ, Pushalkar S, Wu S, Vogel C, Mahal LK, Ghedin E, Ross T, García-Sastre A, Zhang B. 2024. Patient subtyping analysis of baseline multi-omic data reveals distinct pre-immune states associated with antibody response to seasonal influenza vaccination. Clin Immunol 266:110333. doi:10.1016/j.clim.2024.110333 39089348 PMC11340208

[25] Moncunill G, Carnes J, Chad Young W, Carpp L, De Rosa S, Campo JJ, Nhabomba A, Mpina M, Jairoce C, Finak G, et al.. 2022. Transcriptional correlates of malaria in RTS,S/AS01-vaccinated African children: a matched case-control study. Elife 11:e70393. doi:10.7554/eLife.70393 35060479 PMC8782572

[26] Moncunill G, Mpina M, Nhabomba AJ, Aguilar R, Ayestaran A, Sanz H, Campo JJ, Jairoce C, Barrios D, Dong Y, Díez-Padrisa N, Fernandes JF, Abdulla S, Sacarlal J, Williams NA, Harezlak J, Mordmüller B, Agnandji ST, Aponte JJ, Daubenberger C, Valim C, Dobaño C. 2017. Distinct helper T cell type 1 and 2 responses associated with malaria protection and risk in RTS,S/AS01E vaccinees. Clin Infect Dis 65:746–755. doi:10.1093/cid/cix429 28505356 PMC5850568

[27] Diray-Arce J, Miller HER, Henrich E, Gerritsen B, Mulè MP, Fourati S, Gygi J, Hagan T, Tomalin L, Rychkov D, Kazmin D, Chawla DG, Meng H, Dunn P, Campbell J, Sarwal M, Tsang JS, Levy O, Pulendran B, Sekaly R, Floratos A, Gottardo R, Kleinstein SH, Suárez-Fariñas M, Human Immunology Project Consortium (HIPC). 2022. The Immune Signatures data resource, a compendium of systems vaccinology datasets. Sci Data 9:635. doi:10.1038/s41597-022-01714-7 36266291 PMC9584267

[28] Goodwin K, Viboud C, Simonsen L. 2006. Antibody response to influenza vaccination in the elderly: a quantitative review. Vaccine (Auckl) 24:1159–1169. doi:10.1016/j.vaccine.2005.08.105 16213065

[29] Thakar J, Mohanty S, West AP, Joshi SR, Ueda I, Wilson J, Meng H, Blevins TP, Tsang S, Trentalange M, Siconolfi B, Park K, Gill TM, Belshe RB, Kaech SM, Shadel GS, Kleinstein SH, Shaw AC. 2015. Aging-dependent alterations in gene expression and a mitochondrial signature of responsiveness to human influenza vaccination. Aging (Milano) 7:38–52. doi:10.18632/aging.100720 PMC4356402 25596819

[30] Chambers C, Skowronski DM, Rose C, Serres GD, Winter A-L, Dickinson JA, Jassem A, Gubbay JB, Fonseca K, Drews SJ, Charest H, Martineau C, Petric M, Krajden M. 2018. Should sex be considered an effect modifier in the evaluation of influenza vaccine effectiveness? Open Forum Infect Dis 5:fy211. doi:10.1093/ofid/ofy211 PMC6143149 30263903

[31] Tadount F, Kiely M, Assi A, Rafferty E, Sadarangani M, MacDonald SE, Quach C. 2024. Sex differences in the immunogenicity and efficacy of seasonal influenza vaccines: a meta-analysis of randomized controlled trials. Open Forum Infect Dis 11:ofae222. doi:10.1093/ofid/ofae222 38737434 PMC11088355

[32] Kurupati R, Kossenkov A, Haut L, Kannan S, Xiang Z, Li Y, Doyle S, Liu Q, Schmader K, Showe L, Ertl H. 2016. Race-related differences in antibody responses to the inactivated influenza vaccine are linked to distinct pre-vaccination gene expression profiles in blood. Oncotarget 7:62898–62911. doi:10.18632/oncotarget.11704 27588486 PMC5325335

[33] Poland GA, Ovsyannikova IG, Jacobson RM. 2008. Immunogenetics of seasonal influenza vaccine response. Vaccine (Auckl) 26 Suppl 4:D35–40. doi:10.1016/j.vaccine.2008.07.065 PMC2610683 19230157

[34] Brodin P, Jojic V, Gao T, Bhattacharya S, Angel CJL, Furman D, Shen-Orr S, Dekker CL, Swan GE, Butte AJ, Maecker HT, Davis MM. 2015. Variation in the human immune system is largely driven by non-heritable influences. Cell 160:37–47. doi:10.1016/j.cell.2014.12.020 25594173 PMC4302727

[35] Lefkowitz RB, Miller CM, Martinez-Caballero JD, Ramos I. 2024. Epigenetic control of innate immunity: consequences of acute respiratory virus infection. Viruses 16:197. doi:10.3390/v16020197 38399974 PMC10893272

[36] Wimmers F, Donato M, Kuo A, Ashuach T, Gupta S, Li C, Dvorak M, Foecke MH, Chang SE, Hagan T, De Jong SE, Maecker HT, van der Most R, Cheung P, Cortese M, Bosinger SE, Davis M, Rouphael N, Subramaniam S, Yosef N, Utz PJ, Khatri P, Pulendran B. 2021. The single-cell epigenomic and transcriptional landscape of immunity to influenza vaccination. Cell 184:3915–3935. doi:10.1016/j.cell.2021.05.039 34174187 PMC8316438

[37] Apps R, Biancotto A, Candia J, Kotliarov Y, Perl S, Cheung F, Farmer R, Mulè MP, Rachmaninoff N, Chen J, et al.. 2024. Acute and persistent responses after H5N1 vaccination in humans. Cell Rep 43:114706. doi:10.1016/j.celrep.2024.114706 39235945 PMC11949244

[38] Kazmin D, Clutterbuck EA, Napolitani G, Wilkins AL, Tarlton A, Thompson AJ, Montomoli E, Lapini G, Bihari S, White R, Jones C, Snape MD, Galal U, Yu LM, Rappuoli R, Del Giudice G, Pollard AJ, Pulendran B. 2023. Memory-like innate response to booster vaccination with MF-59 adjuvanted influenza vaccine in children. NPJ Vaccines 8:100. doi:10.1038/s41541-023-00702-1 37443176 PMC10344887

[39] Thistlethwaite W, Vangeti S, Cheng W-S, Agarwal P, Cappuccio A, Wang W, Wei B, Myers R, Rubenstein AB, Chawla D, Hariharan M, McClain MT, Burke T, Kleinstein SH, Ecker JR, Woods C, Greenleaf W, Chen X, Ramos I, Zaslavsky E, Evans TG, Troyanskaya OG, Sealfon SC. 2024. Innate immune epigenomic landscape following controlled human influenza virus infection. bioRxiv. doi:10.1101/2024.09.20.612974:2024.09.20.612974 40966076

[40] Mao W, Miller CM, Nair VD, Ge Y, Amper MAS, Cappuccio A, George M-C, Goforth CW, Guevara K, Marjanovic N, et al.. 2023. A methylation clock model of mild SARS-CoV-2 infection provides insight into immune dysregulation. Mol Syst Biol 19:e11361. doi:10.15252/msb.202211361 36919946 PMC10167476

[41] Sparks R, Lau WW, Liu C, Han KL, Vrindten KL, Sun G, Cox M, Andrews SF, Bansal N, Failla LE, et al.. 2023. Influenza vaccination reveals sex dimorphic imprints of prior mild COVID-19. Nature 614:752–761. doi:10.1038/s41586-022-05670-5 36599369 PMC10481789

[42] Hagan T, Cortese M, Rouphael N, Boudreau C, Linde C, Maddur MS, Das J, Wang H, Guthmiller J, Zheng N-Y, et al.. 2019. Antibiotics-driven gut microbiome perturbation alters immunity to vaccines in humans. Cell 178:1313–1328. doi:10.1016/j.cell.2019.08.010 31491384 PMC6750738

[43] Fu H, Pickering H, Rubbi L, Ross TM, Zhou W, Reed EF, Pellegrini M. 2024. The response to influenza vaccination is associated with DNA methylation-driven regulation of T cell innate antiviral pathways. Clin Epigenetics 16:114. doi:10.1186/s13148-024-01730-x 39169387 PMC11340180

[44] Fu H, Pickering H, Rubbi L, Ross TM, Reed EF, Pellegrini M. 2024. Longitudinal analysis of influenza vaccination implicates regulation of RIG-I signaling by DNA methylation. Sci Rep 14:1455. doi:10.1038/s41598-024-51665-9 38228690 PMC10791625

